# Domain-Adaptive Direction of Arrival (DOA) Estimation in Complex Indoor Environments Based on Convolutional Autoencoder and Transfer Learning

**DOI:** 10.3390/s25102959

**Published:** 2025-05-08

**Authors:** Lingyu Shen, Jianfeng Li, Jingjing Pan, Junpeng Shi, Rui Xu, Hao Wang, Weiming Deng

**Affiliations:** College of Electronic and Information Engineering, Nanjing University of Aeronautics and Asteonautics, Nanjing 211106, China; shenlingyu@nuaa.edu.cn (L.S.); jingjingpan@nuaa.edu.cn (J.P.); shijunpeng20@nudt.edu.cn (J.S.); rey.xu@nuaa.edu.cn (R.X.); wanghao319@nuaa.edu.cn (H.W.); dengweiming@nuaa.edu.cn (W.D.)

**Keywords:** indoor scenes, DOA, Deep Learning, domain adaptation

## Abstract

Direction of arrival (DOA) estimation for signal sources in indoor environments has become increasingly important in wireless communications and smart home applications. However, complex indoor conditions, such as multipath effects and noise interference, pose significant challenges to estimation accuracy. This issue is further complicated by domain discrepancies in data collected from different environments. To address these challenges, we propose a deep domain-adaptation-based DOA estimation method. The approach begins with deep feature extraction using a Convolutional Autoencoder (CAE) and employs a Domain-Adversarial Neural Network (DANN) for domain adaptation. By integrating Gradient Reversal Layer (GRL) and Maximum Mean Discrepancy (MMD) loss functions, the model effectively reduces distributional differences between the source and target domains. The CAE-DANN enables transfer learning between data with similar features from different domains. With minimal labeled data from the target domain incorporated into the source domain, the model leverages labeled source data to adapt to unlabeled target data. GRL counters domain shifts, while MMD refines feature alignment. Experimental results show that, in complex indoor environments, the proposed method outperforms other methods in terms of overall DOA prediction performance in both the source and target domains. This highlights a robust and practical solution for high-precision DOA estimation in new environments, requiring minimal labeled data.

## 1. Introduction

In modern life and technological applications, Location-Based Services (LBSs) have become a crucial foundation driving numerous advancements. With the rapid development of smart homes, Virtual Reality (VR), Augmented Reality (AR), and intelligent security systems, indoor positioning technologies have become particularly critical in supporting these emerging technologies [1,2]. Among these, Direction of Arrival (DOA) estimation serves as a common positioning method, providing effective support in specific scenarios. Through DOA estimation, the location and direction of signal sources can be precisely determined, enabling the accurate positioning and dynamic tracking of sound sources, signals, or devices. In some aspects, indoor DOA estimation is even more significant than traditional DOA estimation techniques. By utilizing spatially distributed array sensors, DOA estimation allows devices to intelligently respond to user needs. For example, DOA technology is crucial in healthcare, enabling real-time monitoring and emergency responses by tracking the movements of elderly or vulnerable individuals [3]. While indoor DOA estimation has extensive significance and broad application potential, it also presents unique technical challenges compared to outdoor environments. Traditionally, DOA estimation has been primarily applied in outdoor environments, such as radar and radio positioning. These scenarios typically involve signals following direct paths with minimal interference, allowing for relatively stable and accurate results in short-range distances. However, as the distance increases, even minor angular estimation errors can result in significant positioning inaccuracies when using DOA estimation methods. In contrast, indoor environments, due to their enclosed and complex structures, introduce significant multipath effects [4], with obstacles like walls, furniture, and people causing signal reflection and diffraction. These multipath signals, which vary in phase and amplitude [5], can introduce substantial errors in DOA estimation and may even lead to misdirection.

Classical DOA estimation methods [6], including Beamforming [7,8], subspace decomposition techniques (like MUSIC and ESPRIT) [9,10,11], and compressed sensing approaches based on sparse representation [12,13], are well suited for ideal environments. These methods are widely applied in array signal processing. However, under complex indoor conditions—marked by multipath effects, dynamic interference, and varying channel conditions—traditional methods struggle to maintain robustness in low Signal-to-Noise Ratio (SNR) scenarios [14]. Researchers have developed DOA methods tailored to indoor complexities. To address indoor-specific challenges, researchers have introduced various improvements to traditional DOA methods, enhancing performance amid multipath and dynamic interference. Chahbi and Jouaber investigated a combined Angle of Arrival (AOA), Angle of Departure (AOD), and multipath delay estimation approach, leveraging beamforming techniques to improve precision and mitigate multipath interference; however, performance can degrade in dense multipath environments [15]. Kaneko et al. [16] investigated modifications of the MUSIC algorithm that use iterative techniques to handle coherent and multipath signals, enhancing accuracy and robustness; however, these modifications introduce high computational complexity and sensitivity to low SNRs. V. D [17] proposed a novel DOA method that utilizes manifold reconstruction combined with single-element ESPRIT to address the limitations of traditional approaches, particularly in multipath interference and low SNRs; however, its effectiveness is structure-dependent and lacks robustness in rapidly changing channels. Bhargav et al. [18] developed a compressed sensing-based method that enhances DOA estimation in multipath conditions, utilizing Orthogonal Matching Pursuit (OMP) for sparse signal reconstruction; however, the reconstruction stability of OMP remains a challenge. Bazzi et al. [19] used compressed sensing for DOA estimation with multiple snapshots and works for coherent sources. Hoang et al. [20] proposed a low-complexity compressed sensing technique with a Lens Antenna Array (LAA) that improves precision and reduces computational load under multipath conditions; however, it is inherently dependent on the characteristics of the LAA. For 5G indoor localization, the Joint Angle and Delay Estimation (JADE) approach, combining DOA and Time of Arrival (TOA) estimation, adapts well to GPS-limited indoor environments [21]. In addition, Antonello and colleagues [22] designed a fully hardware-based positioning system utilizing the I/Q method, which performs DOA estimation through phase interferometry.

Recently, machine learning has shown promise in addressing signal processing issues [23], with some research extending it to DOA estimation [24,25,26]. In traditional machine learning, Malajner et al. [26] introduced a DOA method based on Received Signal Strength Indicator (RSSI) and Support Vector Machine (SVM), utilizing RSSI data to predict direction. Salvati [27] proposed an algorithm based on SVM to select the narrow-band components that make positive contributions for DOA estimation. However, these approaches struggle in multipath environments and face challenges like overfitting and the “curse of dimensionality” [28]. Compared to traditional machine learning, Deep Learning (DL) autonomously learns high-level data features from raw inputs, achieving superior predictive performance. Existing DL-based DOA methods typically use received signal data as input and produce corrected signals or directional predictions [29,30]. Some teams have proposed DOA estimation using Deep Neural Networks (DNNs) [31,32], training networks with hidden layers to classify target DOAs within angular grids. Chen et al. [33] developed sector-based DOA estimation networks, reducing computational time by dividing detection and estimation tasks. DL is also used to enhance traditional methods, such as the DeepMUSIC approach by Elbir [34], which uses a Convolutional Neural Network (CNN) to improve MUSIC spectrum peak reconstruction, reducing computation and increasing precision. Xiang et al. [35] introduced an advanced a de-multipath neural network model designed to improve the accuracy of DOA estimation in multipath environments, addressing the challenges posed by signal interference. Similarly, Yu et al. [36] developed a residual Convolutional Neural Network model aimed at model order estimation amid multipath interference, with the objective of enhancing signal processing performance in wireless communication systems.

Despite their potential, DL approaches in DOA estimation are challenged by the reliance on labeled data, which is particularly demanding in indoor environments due to scene complexity, pronounced multipath effects, and varying channel conditions. Consequently, reducing dependency on labeled data while enhancing model adaptability across indoor scenarios remains a key focus in indoor DOA research. Addressing this, we propose a domain-adaptation-based DOA estimation algorithm. The algorithm constructs a labeled source domain dataset by collecting and annotating data in a specific indoor environment. Simultaneously, unlabeled data are collected from another indoor environment, with a small subset of samples annotated and incorporated into the source domain. The remaining data serves as the unlabeled target domain dataset. By leveraging domain adaptation techniques, the algorithm facilitates the transfer of source domain features, significantly reducing distributional differences between the source and target domains. This approach enhances the model’s generalization capability and classification performance in the target domain.

This paper presents a CAE-DANN—an indoor DOA estimation algorithm that combines the benefits of Convolutional Autoencoder (CAE) and Domain-Adversarial Neural Network (DANN), incorporating techniques like the Gradient Reversal Layer (GRL) and Maximum Mean Discrepancy (MMD) to tackle challenges such as data dependency and poor environmental adaptability in indoor DOA estimation. The CAE-DANN extracts deep features from the source domain and applies cross-domain adaptation, enabling efficient and robust DOA estimation across environments. The main contributions are as follows:

1 CAE Model Training and Deep Feature Extraction. A CAE is initially trained on the source domain dataset, leveraging its dimensionality reduction and feature extraction capabilities. CAE’s encoder compresses high-dimensional input into low-dimensional representations, effectively mitigating multipath effects and extracting directional features critical for DOA, enhancing resilience in complex indoor settings.
2 Domain Adaptation Training and Cross-Domain Feature Transfer. Domain adaptation is implemented through adversarial training and self-supervised learning to align deep features from the source to the target domain. During training, supervised learning on the source domain ensures accurate direction representation, while unsupervised learning aligns target data features through adversarial training, minimizing domain distribution discrepancies and enabling cross-domain feature transfer.
3 Dataset Construction and Experimental Evaluation. Separate simulation datasets and measured datasets were constructed. A simplified multipath model is constructed to simulate and generate data with different SNRs in different environments, obtaining multiple sets of datasets. Regarding the measured data, datasets were constructed by collecting and annotating DOA data in a typical indoor environment to create the labeled source domain dataset. Additional DOA data were collected from another indoor environment, with a small portion annotated and added to the source domain, while the rest formed the unlabeled target domain dataset. Experiments on the constructed datasets confirmed the effectiveness of the proposed CAE-DANN.

The paper is divided into five sections. The remaining sections are arranged as follows: Section 2 constructs a uniform circular array (UCA) model and analyzes the reception characteristics of single-source narrowband signals. Section 3 proposes the DOA estimation framework and model design, focusing on the architecture and loss function design of the CAE and DANN. Section 4 describes the experimental setups and data processing procedures for simulation and measurement, demonstrating experimental performance through visual analysis and comparative results. Finally, Section 5 summarizes the advantages of the proposed method in achieving efficient DOA estimation with a small amount of labeled data and discusses its future research and application prospects.

## 2. Model

### 2.1. Array Model

**Figure 1:**
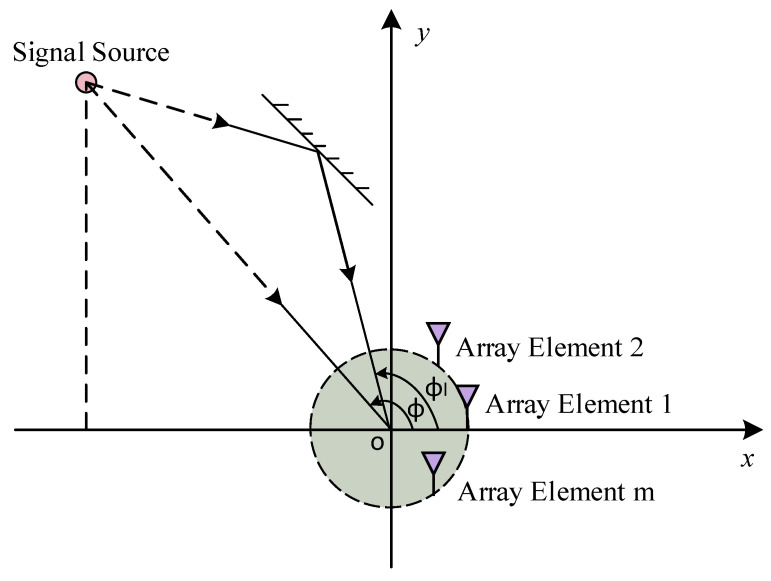
Uniform circular array.

An array model necessary for DOA estimation has been constructed based on a uniform circular array. The array comprises *M* identical omnidirectional antenna elements uniformly distributed along a circle of radius *R* on the x-y plane, as depicted in Figure 1. To represent the direction of the incoming plane wave, a spherical coordinate system is employed, with its origin *O* located at the center of the array, i.e., the center of the circle. The azimuth angle ϕ represents the angle between the line-of-sight (LOS) signal from the source to the origin and the *x*-axis. Similarly, $\phi_{l}$ denotes the angle between the *x*-axis and the *l*-th non-line-of-sight (NLOS) signal that reaches the origin after reflecting off a surface.

The steering vector a(ϕ) represents the array response as a function of ϕ and can be expressed as follows:

(1) $$a\left(\phi \right)=\left[\begin{array}{c} \exp\left(j2\pi R\frac{\cos(\phi -\gamma_{0})}{\lambda }\right)\\ \exp\left(j2\pi R\frac{\cos(\phi -\gamma_{1})}{\lambda }\right)\\ \vdots \\ \exp\left(j2\pi R\frac{\cos(\phi -\gamma_{M-1})}{\lambda }\right) \end{array}\right]$$

where *R* denotes the radius, λ denotes the wavelength of the signal, $\gamma_{m}=2\pi m/M$, m=0,1,⋯,M−1.

### 2.2. Signal Model

This paper focuses exclusively on the single-source scenario. Assume there is a narrowband signal source with a complex amplitude impinging on a UCA of *M* elements from direction ϕ. Then, the received signal at time *t* is

(2) x(t)=a(ϕ)s(t)+n(t),t=1,2,⋯,Ns

where the received signal $x\left(t\right)={\left[x_{1}\left(t\right),x_{2}\left(t\right),\cdots ,x_{M}\left(t\right)\right]}^{T}\in C^{M\times 1}$. a(ϕ) is the corresponding steering vector. s(t) represents the incident signal matrix from a single signal source, while $n\left(t\right)={\left[n_{1}\left(t\right),n_{2}\left(t\right),\cdots ,n_{M}\left(t\right)\right]}^{T}\in C^{M\times 1}$ denotes the noise matrix, with each noise vector being independently distributed. Additionally, $n_{i}\left(t\right)∼N\left(0,\sigma^{2}\right),\;i=1,2,\cdots ,M$, and $\sigma^{2}$ represent the noise power. Ns is the number of snapshots.

Furthermore, if multipath signals are present in the environment, let *L* denote the number of multipaths. In this case, the received signal can be reformulated as the sum of the multipath signals:

(3) $$x\left(t\right)=\sum_{l=0}^{L}a_{l}s\left(t-\tau_{l}\right)+n_{l}\left(t\right)$$

where $\tau_{l}$ represents the time delay of the *l*-th propagation path, with l=0 denoting the LOS path and $a_{l}$, $s(t-\tau_{l})$, and $n_{l}\left(t\right)$ respectively represent the corresponding steering vector, signal, and noise corresponding to the *l*-th path. The NLOS signals caused by multipath, along with the LOS signal, will form coherent signals that affect the DOA estimation.

To obtain DOA information, calculating the covariance matrix of the received signal is an effective approach. Taking the case without the multipath as an example, and without loss of generality, the time index t can be ignored. We assume that all signal and noise components are uncorrelated and that the noise components have the same power. Thus, the covariance matrix of the received signal, denoted by $R_{x}\in C^{M\times M}$, is given by:

(4) $$R_{x}≜E\left[x\left(t\right)x^{H}\left(t\right)\right]=a\left(\phi \right)R_{s}a^{H}\left(\phi \right)+R_{n}$$

where $R_{s}$ denotes the covariance matrix of the signal, while $R_{n}$ represents the covariance matrix of the noise.

In practical situations, it is not feasible to obtain the probabilistic mean based on the definition; hence, an arithmetic mean is calculated to approximate the covariance matrix of the received signal:

(5) $$R_{x}=E\left[x\left(t\right)x{\left(t\right)}^{H}\right]\approx \frac{1}{Ns}x\left(t\right)x{\left(t\right)}^{H}$$

The covariance matrix of the received signal contains a lot of information, and the amount of data is greatly reduced compared with the received signal itself. As an important tool to analyze the received signal, the covariance matrix is used as the input of our network to extract the features from the received signals.

## 3. The CAE-DANN Network

### 3.1. Framework for the DOA Estimation Process

**Figure 2:**
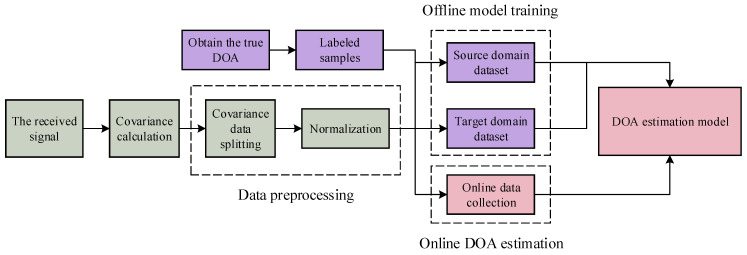
Framework for the DOA estimation process.

The estimation framework consists of three phases: dataset collection, offline training, and online estimation, as illustrated in Figure 2. During the data collection phase, data must be obtained from at least two indoor environments. One environment is used to construct the labeled source domain dataset, while the others are used to build the unlabeled target domain dataset. (To mitigate inter-domain distribution differences, a small number of samples from the target domain must be annotated and incorporated into the source domain.) A large number of array-received signals from different angles are collected through either simulation or actual measurements. The offline training phase involves training the model using a set of source domain data and a set of target domain data. In the online estimation phase, collected received signal samples are input for DOA estimation.

In the estimation phase, the model uses the signal covariance matrix as input for the source data, followed by data preprocessing to create the dataset. Specifically, (1) to facilitate processing by the Deep Learning model and to fully utilize the information from the covariance matrix, the complex matrix is divided into three components: real part, imaginary part, and phase; (2) to ensure that the sample matrices have a unified standard for operations such as dot products or other calculations, normalization is performed. The model is trained using a semi-supervised learning approach. For the source domain dataset, in addition to processing the received signals as described, the corresponding DOA of the LOS path is also input as label data, with domain labels of 0 for the source domain dataset and 1 for the target domain dataset.

### 3.2. Dataset Construction

To construct a dataset for DOA estimation in indoor environments, we extract features from the covariance matrix of the received signals to estimate the DOA. To preserve the spatial structure and correlations of the data, facilitating the model’s ability to capture relationships among features, we maintain the complete covariance matrix and decompose it into three separate matrices: the real part, the imaginary part, and the phase. These matrices are then normalized using the min-max normalization method, ensuring that the data are on a consistent scale. This approach enhances numerical stability and accelerates network convergence, ultimately resulting in a three-channel data matrix D to be used as input:

(6) Re˜(Rx)=Re(Rx)−min(Re(Rx))max(Re(Rx))−min(Re(Rx))

(7) Im˜(Rx)=Im(Rx)−min(Im(Rx))max(Im(Rx))−min(Im(Rx))

(8) $$P\tilde{has}e\left(R_{x}\right)=\frac{Phase\left(R_{x}\right)-\min\left(Phase\left(R_{x}\right)\right)}{\max\left(Phase\left(R_{x}\right)\right)-\min\left(Phase\left(R_{x}\right)\right)}$$

(9) D=[Re˜(Rx)Im˜(Rx)Phas˜e(Rx)]

where Re(·), Im(·) and Phase(·) represent the extracted real part, imaginary part, and phase of the complex matrix, respectively.

The predicted DOA results serve as the output Y of the label predictor, while the predicted domain category serves as the output *Z* of the domain classifier. Together, the predicted DOA and domain information constitute the network’s output, establishing the input–output relationship {D,Y,Z} for the network (with Y excluded for the target domain). Specifically, for source domain data, the corresponding DOA information Y and domain label Z=0 are used as label data, while for target domain data, only the domain label Z=1 is used. Here, $D\in C^{3\times M\times M}$, $Y\in R^{K\times 1}$, and Z∈{0,1}.

### 3.3. Model Design and Training

#### 3.3.1. Overall Network Model

**Figure 3:**
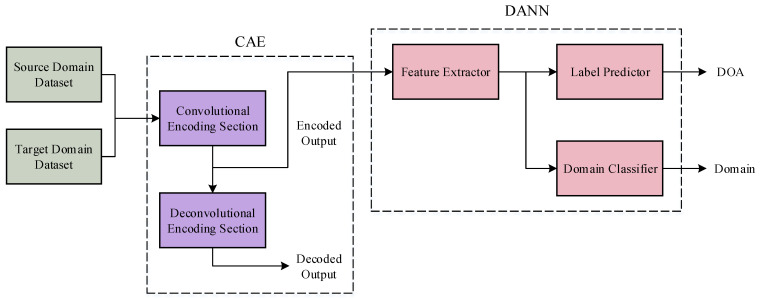
The overall network architecture of CAE-DANN.

After constructing the dataset, a DOA estimation model was developed, as shown in Figure 3. This model comprises two primary components: a CAE and a DANN. Initially, both the source domain and target domain datasets are input into the CAE, which encodes the covariance matrices of the received signals. The encoded representations are subsequently passed to the DANN to facilitate domain adaptation between the source and target domains. As a result, the network effectively estimates DOA information with high accuracy across both domains.

#### 3.3.2. Convolutional Autoencoder

Due to the presence of significant multipath effects and noise in actual signals, the CAE effectively extracts and compresses essential features from the covariance matrices that contain these multipath effects. This process reduces noise and redundant information, resulting in a low-dimensional representation. The encoded features are then fed into the DANN, enabling the model to learn features that are insensitive to variations across different data domains. Ultimately, this enhances the accuracy of DOA estimation.

**Figure 4:**
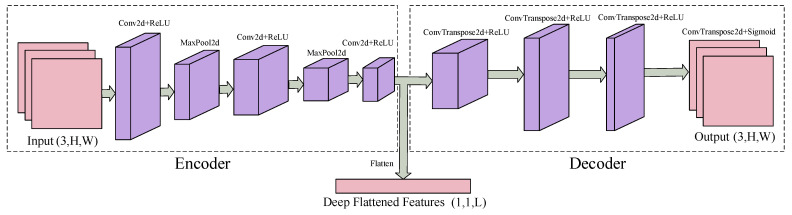
The specific structure of CAE.

The CAE is a deep self-encoding model based on CNN, consisting primarily of an encoder and a decoder [37]. The encoder includes convolutional layers, pooling layers, and activation functions, which are responsible for compressing and extracting features from the input data. The decoder is a mirror of the encoder, comprising deconvolutional layers and activation functions, tasked with decoding and reconstructing the encoded data. As shown in Figure 4, the CAE network features eight hidden layers, with a flattening layer that transforms the multi-dimensional feature maps compressed by the encoder into a one-dimensional vector for further processing by the DANN.

The specific processing procedure of the CAE network is as follows. In the first convolution, the input is the preprocessed covariance matrix $D^{\left(u\right)}$, with dimensions 3×M×M. The size of the convolutional kernel W is w×w, and there are *k* convolutional kernels. The output feature of the *k*-th convolutional kernel is

(10) $$h^{k}=\sigma \left(D^{\left(u\right)}*W^{k}+b^{k}\right)$$

where *b* represents the bias for each layer, and σ is the activation function. In this experiment, ReLU is chosen as the activation function, while ∗ denotes the convolution operation. The three convolution operations follow the same procedure, differing only in the size and number of convolutional kernels. Between every two convolutions, there is also a max-pooling layer for further dimensionality reduction, ultimately yielding the encoded output $F^{\left(u\right)}$. This $F^{\left(u\right)}$ is then decoded and reconstructed to obtain the decoded output ${\hat{D}}^{\left(u\right)}$. The decoding is performed through deconvolution, with the deconvolution layer’s process defined as follows:

(11) $$h^{k*}=\sigma \left(\sum_{k\in H}F^{(u)k}*{\tilde{W}}^{k}+c^{k}\right)$$

where c is the bias term for the deconvolution, h* is the output of the deconvolution, and $\tilde{W}$ represents the convolutional kernels used in the deconvolution process. After passing through four subsequent deconvolution layers, the decoded output ${\hat{D}}^{\left(u\right)}$ is obtained. The cost function of this network is defined as the Mean Squared Error (MSE):

(12) $$L_{CAE}=\frac{1}{U}\sum_{i=1}^{U}{\left(D^{\left(u\right)}-{\hat{D}}^{\left(u\right)}\right)}^{2}$$

where *U* represents the total number of samples.

Finally, the backpropagation algorithm is employed to compute the gradients of the cost function, allowing for the adjustment of the neural network’s weights and biases. Upon completion of the model training, the preprocessed covariance matrix can be input to obtain the encoded features.

#### 3.3.3. Domain Adversarial Neural Network

The target domain dataset is entirely unlabeled, presenting a more challenging unsupervised learning scenario. In the case where there is a distribution shift between the source and target domains, we employ DANN [38,39], complemented by MMD [40,41], to mitigate the distributional discrepancy between the source and target domains. This approach enables the network to effectively perform DOA estimation on the target domain data.

**Figure 5:**
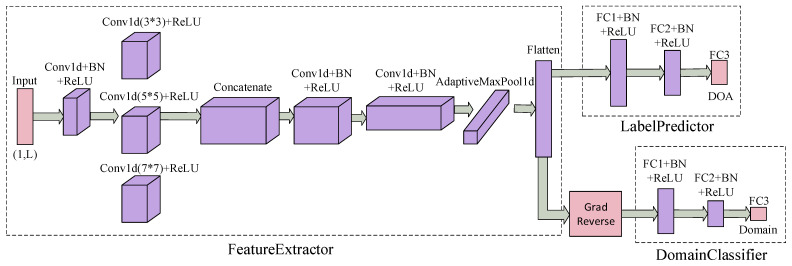
The specific structure of DANN.

In DANN, the architecture consists of three main components: a feature extractor, a label predictor, and a domain classifier, as illustrated in Figure 5. The network receives as input a source domain dataset with labeled categories and a target domain dataset without labels, along with domain classification labels for both the source and target domains. Since the input to this network module has already undergone deep feature extraction by the CAE, the source domain data distribution S(D,Y) can be transformed into $S(F_{l},Y)$. Similarly, the target domain data distribution T(D,Y) can be transformed into $T(F_{l},Y)$.

During the training phase, the encoded and flattened deep features *a* are mapped into a feature vector *t* by the feature extraction network $G_{f}\left(a;\theta_{f}\right)$. The network then splits into two branches: a label classifier $G_{y}\left(t;\theta_{y}\right)$ and a domain classifier $G_{d}\left(t;\theta_{d}\right)$. The feature vector corresponding to the source domain input is mapped through $G_{y}\left(t;\theta_{y}\right)$ to obtain the respective DOA prediction results. Meanwhile, the feature vectors, regardless of whether they originate from the source or target domain input, pass through $G_{d}\left(t;\theta_{d}\right)$ to yield the domain classification results for each input.

Although DANN can only perform DOA estimation on the source domain data, to enable prediction on the target domain dataset, the network must treat the target domain data as if it were from the source domain. Therefore, during the training phase, two objectives are pursued: (1) accurately predict the source domain dataset, minimizing regression loss; (2) confuse the source domain dataset with the target domain dataset, thereby maximizing regression loss.

To achieve the adversarial learning required for the second task, a GRL is employed between the feature extractor and the domain classifier. This allows the gradients to pass through as an identity transformation during forward propagation while automatically reversing their direction during backpropagation. The GRL can be viewed as a “pseudo-function” $H_{\lambda }\left(x\right)$, with its forward and backward propagation behaviors defined by the following two equations:

(13) $$H_{\lambda }\left(x\right)=x$$

(14) $$\frac{dH_{\lambda }}{dx}=-\lambda I$$

where I represents the identity matrix and λ controls the extent to which the gradients are reversed.

In the GRL, the parameter λ is not a fixed value; instead, it changes dynamically:

(15) $$\lambda_{p}=\frac{2}{1+\exp(-\delta \cdot p)}-1$$

where δ is a constant with a value of 10, and *p* represents the relative progress of iterations, which is the ratio of the current training epoch to the total number of epochs.

Through the analysis of the network structure and the introduction of the GRL, we obtain the label prediction loss $L_{doa}$ and the domain classification loss $L_{domain}$:

(16) $$L_{doa}=\sum_{\begin{array}{c} u=1,\cdots ,U\\ Z=0 \end{array}}L_{y}\left(G_{y}\left(G_{f}\left(F_{l}^{\left(u\right)};\theta_{f}\right);\theta_{y}\right),Y^{\left(u\right)}\right)$$

(17) $$L_{domain}=\sum_{u=1,\cdots ,U}L_{d}(G_{d}\left(H_{\lambda }\left(G_{f}\left(F_{l}^{\left(u\right)};\theta_{f}\right);\theta_{d}\right),Y^{\left(u\right)}\right)$$

where the label prediction loss $L_{doa}$ employs the Root Mean Squared Error (RMSE) loss function $L_{y}$, while the domain classification loss $L_{domain}$ utilizes the binary cross-entropy loss function $L_{d}$.

The RMSE loss function used by $L_{doa}$ is expressed as follows:

(18) $$L_{\mathrm{doa}}=\sqrt{\frac{1}{U}\sum_{u=1}^{U}{\left({\hat{Y}}_{u}-Y_{u}\right)}^{2}}$$

where ${\hat{Y}}_{i}$ and $Y_{i}$ represent the predicted value and the ground truth of the *u*-th DOA data, respectively.

To make the distributions of the two datasets more similar, we also introduce MMD. MMD is used to quantify the difference between the probability distributions of the source domain *S* and the target domain *T*. The core idea is to map the data into a high-dimensional feature space using a kernel function and then compute the mean discrepancy between the two distributions in that space. It is defined as

(19) $$L_{MMD}={∥\frac{1}{m}\sum_{i=1}^{m}\phi \left(F_{l,Z=0}^{\left(i\right)}\right)-\frac{1}{n}\sum_{j=1}^{n}\phi (F_{l,Z=1}^{\left(j\right)})∥}_{H}^{2}$$

where $F_{l,Z=0}^{\left(i\right)}$ represents *m* samples drawn from the source domain distribution *S*, and $F_{l,Z=1}^{\left(j\right)}$ represents *n* samples drawn from the target domain distribution *T*. The function ϕ(x) is a mapping that projects the samples into a high-dimensional Hilbert space *H*. This mapping is achieved by selecting a kernel function k(x,y), which defines the specific transformation into the feature space.

Expanding and simplifying the above expression yields the estimation formula:

(20) $$L_{MMD}=\frac{1}{m^{2}}\sum_{i=1}^{m}\sum_{j=1}^{m}k\left(F_{l,Z=0}^{\left(i\right)},F_{l,Z=0}^{\left(j\right)}\right)+\frac{1}{n^{2}}\sum_{i=1}^{n}\sum_{j=1}^{n}k(F_{l,Z=1}^{\left(i\right)},F_{l,Z=1}^{\left(j\right)})-\frac{2}{mn}\sum_{i=1}^{n}\sum_{j=1}^{n}k(F_{l,Z=0}^{\left(i\right)},F_{l,Z=1}^{\left(i\right)})$$

where k(x,y) is the kernel function, and we select the Gaussian kernel function, defined as

(21) $$k\left(x,y\right)=\exp\left(-\frac{{∥x-y∥}^{2}}{2\sigma^{2}}\right)$$

where ${∥x-y∥}^{2}$ represents the squared Euclidean distance between samples, and the hyperparameter σ controls the width or smoothness of the Gaussian kernel.

In summary, the GRL and MMD work in close collaboration to achieve domain adaptation and feature alignment. The GRL, by automatically reversing the gradient direction during backpropagation, makes it difficult for the domain classifier to distinguish the feature representations of source and target domain data, encouraging the feature extractor to learn domain-invariant features that are unaffected by the domain of the data. This forms the foundation for domain adaptation. On the other hand, the MMD, which quantifies the distributional differences, maps the source and target domain data into a high-dimensional feature space and computes their average discrepancy in this space. By minimizing the MMD loss, the model further adjusts the feature distribution, making the features of the source and target domains more similar in the high-dimensional space, thereby enhancing the feature alignment. During training, GRL primarily drives the feature extractor to learn shared features from an adversarial training perspective, while MMD fine-tunes the features from the perspective of distributional discrepancy. The two functions work together to reduce the distributional difference between the source and target domains, ultimately improving DOA estimation performance in the target domain.

Therefore, the overall loss function of the network comprises the label prediction loss, domain classification loss, and MMD loss:

(22) $$L_{total}=L_{doa}+\alpha \cdot L_{domain}+\beta \cdot L_{MMD}$$

where the weights α and β correspond to $L_{domain}$ and $L_{MMD}$, respectively.

During the training process, α and β control the weights of $L_{domain}$ and $L_{MMD}$, respectively. A larger α and β will make the model pay more attention to the differences between domains and thus neglect the learning of source domain data. When α and β are relatively small, the model will be unable to effectively align the feature distributions of the source domain and the target domain. The values of α and β are set in a linearly increasing manner. In the early stage of training, $L_{doa}$ dominates, and the model first learns the basic feature of the source domain. As the training progresses, α and β gradually increase, and the model pays more attention to domain alignment, thereby improving its prediction ability in the target domain.

### 3.4. Time Complexity

The complexity of CAE-DANN was evaluated by comparing its average runtime with DANN, CNN, and MUSIC across 100 independent experiments. The experiments were conducted on a system equipped with an Intel(R) Core(TM) i7-9750H CPU and an NVIDIA GeForce GTX 1650 GPU. The results are shown in Table 1. The runtime of CAE-DANN is comparable to that of DANN and CNN, falling within the same order of magnitude and exhibiting relatively low runtime. This indicates that CAE-DANN achieves runtime efficiency on par with mainstream Deep Learning methods. However, due to the additional incorporation of CAE and the optimization of the loss function design, there is a slight increase in computational overhead. In contrast, compared to the traditional signal processing method MUSIC, the runtime of CAE-DANN is significantly reduced. This shorter runtime enables CAE-DANN to perform real-time estimation, making it well suited for applications with stringent real-time requirements.

## 4. Experiment

### 4.1. Simulation Experiment

#### 4.1.1. Simulation Setup

We first verify the advantages of the proposed method in terms of training performance through simulation experiments. Based on the model constructed from Section 2, we further simplify the multipath signal model. In this model, only the LOS signal from a single signal source and the primary multipath components are considered. The attenuation and delay of the LOS signal are ignored, retaining only the attenuation effects caused by reflection while neglecting natural attenuation during propagation to reduce computational complexity. The simplified multipath signal model can be expressed as

(23) $$x\left(t\right)=a\left(\phi \right)s\left(t\right)+\alpha_{1}a\left(\phi_{1}\right)s\left(t-\tau_{1}\right)+n\left(t\right)$$

where ϕ and $\alpha_{1}$ represent the angles of the LOS signal and the primary multipath components, respectively. The parameter $\alpha_{1}$ denotes the attenuation factor of the primary multipath components, while $\tau_{1}$ represents the time delay.

This simplification is reasonable for performance comparison, as data-driven methods, unlike model-driven approaches, do not utilize any prior information about the steering vector.

In the simulation experiment, we generate narrowband radio frequency (RF) signals with a sampling frequency set to 1 MHz. The baseband signal is a 1 kHz sine wave, which is modulated onto a 2.4 GHz carrier to produce both direct and multipath signals. In this simulation environment, the multipath attenuation factors for the source and target domains are set to 4 dB and 5 dB, respectively, while the multipath propagation delays are 50 ns and 60 ns, respectively. Furthermore, we constructed a UCA and uniformly sampled the incident angles of the LOS signal in the range of [0°,360°) at 1° intervals. To more realistically simulate the wireless channel environment, the primary multipath signal angles are generated using a hybrid model that combines mirror reflection and a Gaussian distribution:

(24) $$\phi_{1}=2\phi^{\prime }-\phi +N\left(0,\sigma^{2}\right)$$

where $\phi^{\prime }$ represents the normal angle of the reflective surface, $N(0,\sigma^{2})$ denotes a Gaussian-distributed random variable, and σ is used to model angular perturbations caused by surface roughness. In this experiment, the $\phi^{\prime }$ was randomly chosen from multiple wall orientations at $0^{\circ }$, $90^{\circ }$, $180^{\circ }$, and $270^{\circ }$, with $\sigma =5^{\circ }$, in order to more accurately simulate the angular variations in an indoor multipath environment.

Ultimately, the SNR is uniformly sampled within the range of [−20 dB, 0 dB] at 5 dB intervals. For each SNR level and each angular configuration, 100 corresponding covariance matrices are generated. Consequently, both the source and target domains of the dataset contain a total of 180,000 data samples.

#### 4.1.2. Simulation Results and Analysis

To comprehensively evaluate the network’s performance, we adopted Root Mean Squared Error (RMSE) and Mean Absolute Error (MAE) as evaluation metrics. RMSE penalizes larger errors more heavily, making it sensitive to the model’s performance in handling large deviations, while MAE provides a balanced measure of errors, reflecting the model’s overall prediction accuracy more stably. By simultaneously analyzing these two metrics, we can more comprehensively assess the model’s performance across different error magnitudes.

RMSE is defined as

(25) $$RMSE=\sqrt{\frac{1}{N}\sum_{n=1}^{N}{\left({\hat{z}}_{n}-z_{n}\right)}^{2}}$$

where the sample size is *N*, and ${\hat{z}}_{n}$ is the estimated value of $z_{n}$ in the *n*-th Monte Carlo experiment.

Similarly, MAE is defined as

(26) $$MAE=\frac{1}{N}\sum_{n=1}^{N}|{\hat{z}}_{n}-z_{n}|$$

**Figure 6:**
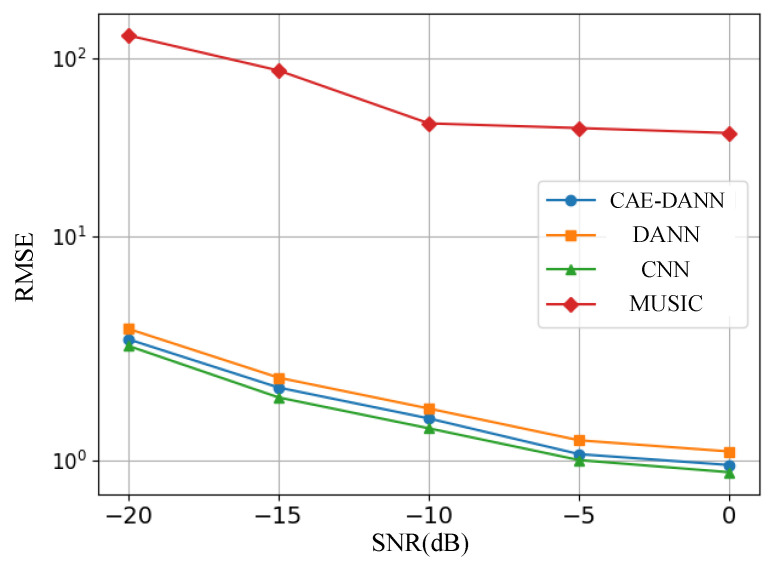
Source domain RMSE of methods.

**Figure 7:**
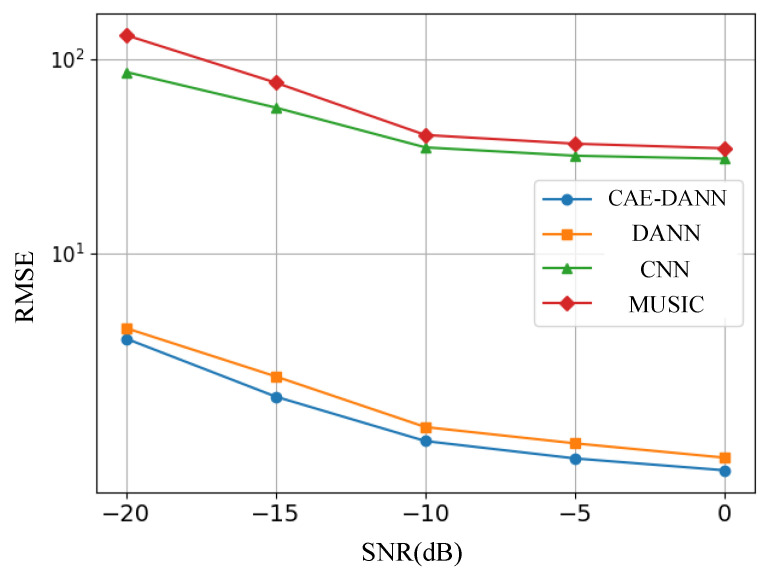
Target domain RMSE of methods.

**Figure 8:**
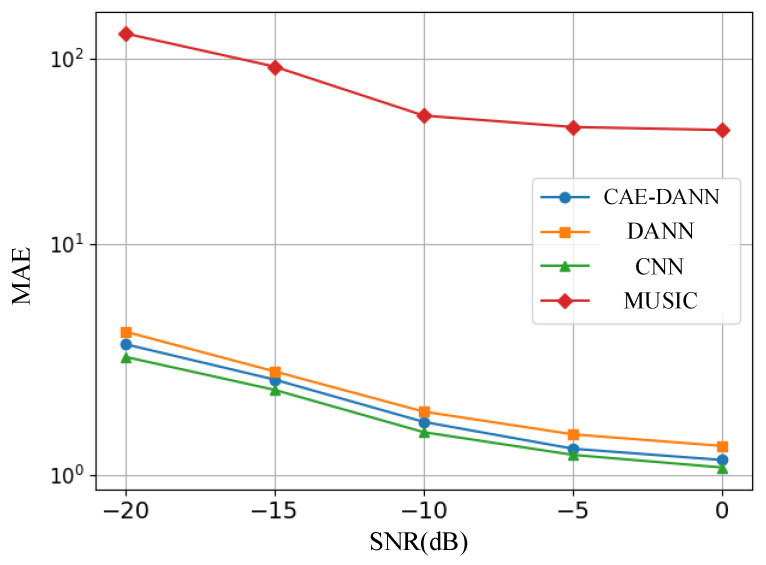
Source domain MAE of methods.

**Figure 9:**
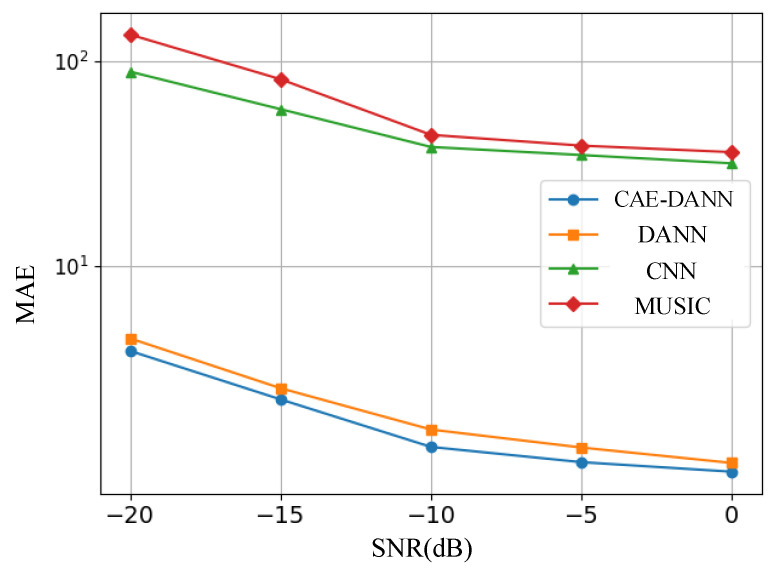
Target domain MAE of methods.

As shown in Figure 6, Figure 7, Figure 8 and Figure 9, through 500 Monte Carlo experiments, the RMSE and MAE of different methods under various SNR conditions are presented. It can be observed from these figures that, compared with other methods, the CAE-DANN consistently shows significantly lower errors across all SNR levels. CAE-DANN demonstrates excellent robustness in noisy environments, especially under low SNR conditions, where it can effectively control the errors and achieve better performance. For MUSIC, these figures show that its error values are relatively high in both the source and target domains. This is mainly because in a multi-path environment, this method is unable to estimate the DOA accurately. In contrast, CAE-DANN, with its feature extraction and domain adaptation capabilities, can effectively cope with the impact of noise and adapt to different SNR conditions, maintaining excellent performance in both the source domain and the target domain. Although CNN achieves lower errors in the source domain, due to the lack of training on the target domain data, its performance in the target domain is relatively poor. In comparison, although DANN has certain cross-domain adaptation capabilities, its effectiveness is not as good as that of CAE-DANN. In addition, by comparing different error metrics, it can be found that RMSE is usually slightly lower than MAE, and their changing trends are consistent. This phenomenon can be attributed to the fact that RMSE is more sensitive to larger errors. CAE-DANN comprehensively considers these two error metrics in different SNR environments and achieves a good balance between controlling larger errors and improving the overall prediction stability.

### 4.2. Measured Experiment

#### 4.2.1. Measured Setup and Instruments

**Figure 10:**
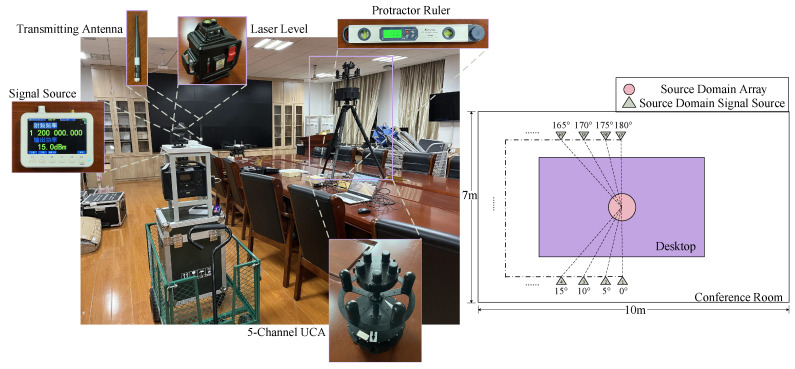
Conference room setting and experimental instruments (source domain).

**Figure 11:**
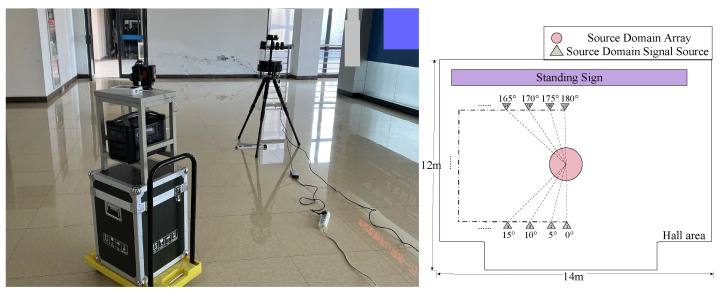
Hall setting (target domain).

The experiment was conducted within a conference room and a hall. In the center of the conference room, depicted in Figure 10, a large conference table was positioned, surrounded by rows of wall-adjacent tables holding a variety of equipment. In the hall, shown in Figure 11, one side featured a large standing sign, while the opposite side included a protruding structure that connected to the elevator, creating a non-standard rectangular shape for the space. These two distinct and complex indoor environments ensured a rich presence of multipath effects and other intricate signal propagation influences, providing a representative and challenging indoor scenario for the experiment.

The experimental instruments are displayed in Figure 10. A portable RF signal source, the SG-3000PRO, was employed for signal transmission, with a frequency range covering 10 MHz to 3 GHz and a maximum output power of +20 dBm. To ensure effective radiation within the specified frequency range, the HT1200LC whip antenna was selected as the transmitting antenna. This antenna, operating across 100 MHz to 1.2 GHz, is suitable for mid- to low-frequency signal transmission, ensuring stable signal propagation.

The receiving array used is a 5-channel direction-finding monitoring system, covering a frequency range of 20 MHz to 5 GHz. Its two-layer array spans progressively from low to high frequencies: 20 MHz to 3 GHz and 3 GHz to 5 GHz. The receiving antennas are vertically polarized, capable of outputting either IQ data segments or continuous signals.

To ensure precise angular calibration, a Delixi laser level and a protractor ruler were utilized during the experiment, minimizing measurement errors as much as possible.

#### 4.2.2. Measured Data Collection

We collected source domain data in a conference room and target domain data in a hall, with a small portion of the hall data incorporated into the source domain. In the experiment, a UCA with five elements was fixed at the center of the scene, and the DOA was varied by moving the signal source, thus enabling the collection of corresponding IQ data. Considering the large amount of data and the potential for errors from various angles, we uniformly collected IQ data at 5° intervals within the range of 0° to 180°, as illustrated in Figure 10 and Figure 11.

During data acquisition, the signal source emitted a narrowband RF signal with a center frequency of 1200 MHz and a power of 15 dBm. The array system was tuned to the same center frequency, with a capture bandwidth set to 6.25 kHz and an effective sampling rate of 12.5 kHz, collecting 2048 samples per snapshot. Using a laser level and a protractor ruler for alignment, the signal source was sequentially positioned at 37 locations corresponding to different DOAs. At each point, 5000 KB of IQ data were collected, resulting in 37 datasets for each scene. Subsequently, 500 snapshots were extracted from each dataset. For the hall target domain, 10 snapshots per location were randomly selected, annotated, and incorporated into the source domain.

#### 4.2.3. Network Training Configuration

The source domain samples were split into training and testing sets at an 8:2 ratio to facilitate model training in later stages. The training of CAE and DANN was conducted sequentially, with the specific hyperparameters detailed in Table 2 and Table 3, respectively.

#### 4.2.4. Measured Results and Analysis

First, we collected and preprocessed datasets from the source and target domains under two distinct scenarios. By applying standardization and normalization techniques, we ensured the uniformity of feature distributions, laying a solid foundation for subsequent model training. After completing the data preprocessing, we conducted offline training based on the aforementioned network configuration, iteratively optimizing model parameters and achieving feature alignment. Ultimately, we successfully trained the CAE-DANN model.

**Figure 12:**
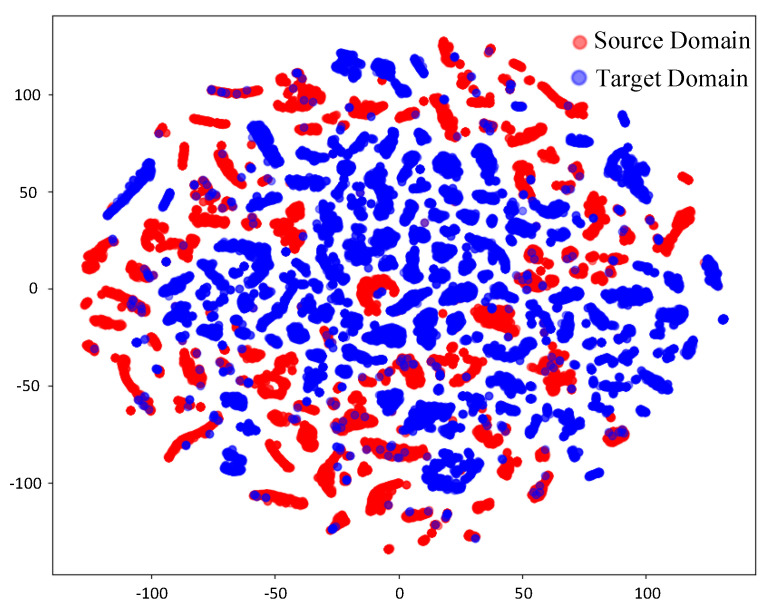
Feature before domain adaptation.

**Figure 13:**
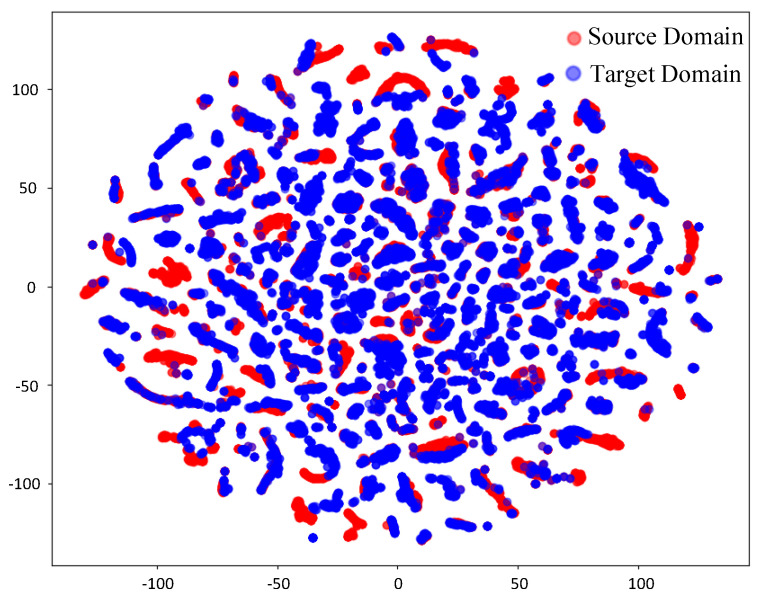
Feature after domain adaptation.

Before domain adaptation and feature alignment (Figure 12), the t-SNE visualization reveals limited overlap between the red points (source domain) and the blue points (target domain), while the overall distributions of the two domains remain distinctly separable. This partial overlap reflects the inclusion of a small amount of hall scene data in the source domain dataset and the presence of shared features in LOS information across different scenarios. However, the pronounced separation of most points highlights the significant disparity in feature distributions between the source and target domains, primarily attributed to the complex effects of NLOS propagation. After domain adaptation and feature alignment (Figure 13), a noticeable overlap emerges between the red points (source domain) and the blue points (target domain), clearly demonstrating the effectiveness of CAE-DANN in achieving feature alignment. The integration of source and target domain features in the distribution is significantly enhanced, indicating that the network has successfully minimized inter-domain discrepancies. This feature distribution convergence greatly improves the model’s generalization ability in the target domain, enabling the classifier to establish more accurate decision boundaries based on the aligned features. Specifically, the GRL component within the network effectively mitigates domain shift through adversarial training, facilitating the learning of domain-invariant shared features. Meanwhile, the MMD metric further reduces distributional discrepancies in the feature space between the source and target domains. This dual mechanism effectively promotes feature alignment across domains. The partial misalignment of features is attributed to the differences caused by complex scenarios, such as multipath effects, scattering, and occlusion, which prevent complete uniformity in feature distributions.

**Figure 14:**
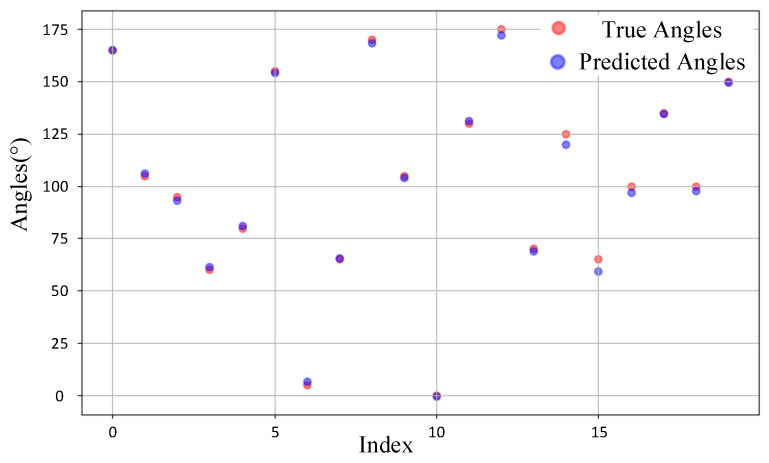
Comparison of source domain samples.

**Figure 15:**
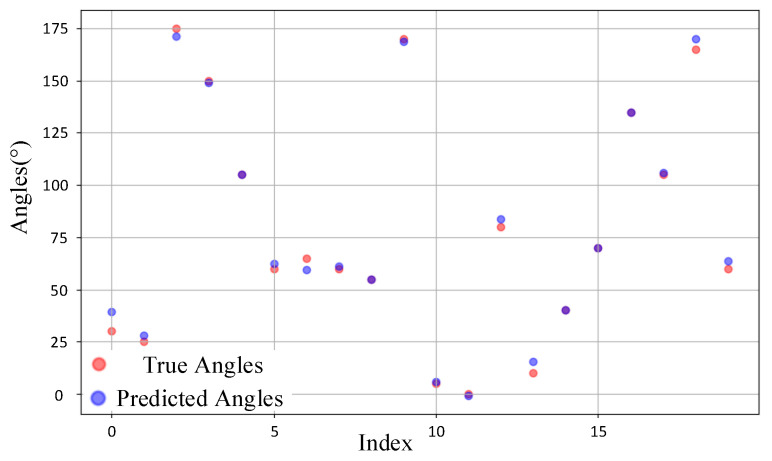
Comparison of target domain samples.

We randomly selected 20 samples from the test sets of the source domain and target domain for observation. Figure 14 illustrates the comparison between the predicted DOA values and the ground truth values for the source domain samples. It can be observed that for most samples, the predicted values align closely with the ground truth, with the majority of points almost overlapping. This indicates that the model demonstrates high prediction accuracy in the source domain. In contrast, Figure 15 depicts the comparison for the target domain samples. While the deviations between predicted and true values are slightly larger compared to the source domain, the overall predictions remain reasonably accurate. This suggests that the model maintains a satisfactory level of performance even when applied to the target domain. The overall accuracy remains at a satisfactory level, indicating that CAE-DANN successfully aligns the feature distributions of the source and target domains even under complex real-world scenarios, providing a robust solution for DOA prediction in the target domain.

To further validate the superiority of CAE-DANN under different domain settings, we conducted 500 Monte Carlo experiments to systematically compare the RMSE and MAE performance of CAE-DANN with those of other methods in both the source and target domains. In addition to comparing with the Deep Learning method CNN and the traditional method MUSIC, we also designed ablation experiments to examine the impact of different parts of the network on its performance. Specifically, all samples were randomly selected from the test datasets of the corresponding domains for the experiments.

As shown in Table 4 and Table 5, CAE-DANN consistently exhibits lower errors in both domains, with results significantly outperforming other methods, particularly in terms of the prediction ability in the target domain. In contrast, although DANN (without CAE), CAE-DANN (without MMD), and CAE-CNN (without GRL) demonstrate certain cross-domain capabilities, their errors in the target domain are higher than those of CAE-DANN. This highlights the effectiveness of CAE in extracting deep features, as well as the enhanced domain adaptation ability achieved through the integration of the GRL and the MMD. By comparing CAE-DANN (without MMD) and CAE-CNN (without GRL), it can be seen that GRL shows stronger cross-domain capabilities than MMD in this network. The feature alignment between the target domain and the source domain sacrifices the prediction ability for the source domain to a certain extent, but it is acceptable. The CNN model performs best in the source domain; however, its error in the target domain surges, revealing its extreme sensitivity to cross-domain distribution shifts and poor adaptability. In contrast, the traditional MUSIC shows high errors in both the source and target domains and performs poorly in complex multipath scenarios. Overall, by incorporating the autoencoder module, CAE-DANN performs excellently in both domains, especially reducing the RMSE and MAE errors in the target domain.

**Figure 16:**
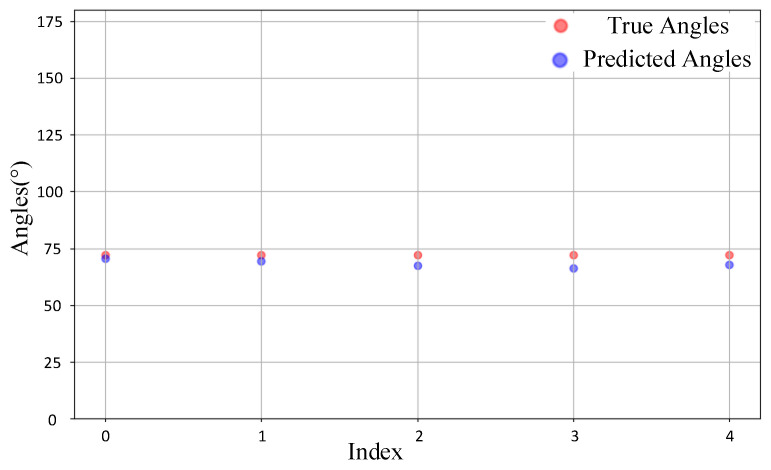
Comparison of 72° samples.

**Figure 17:**
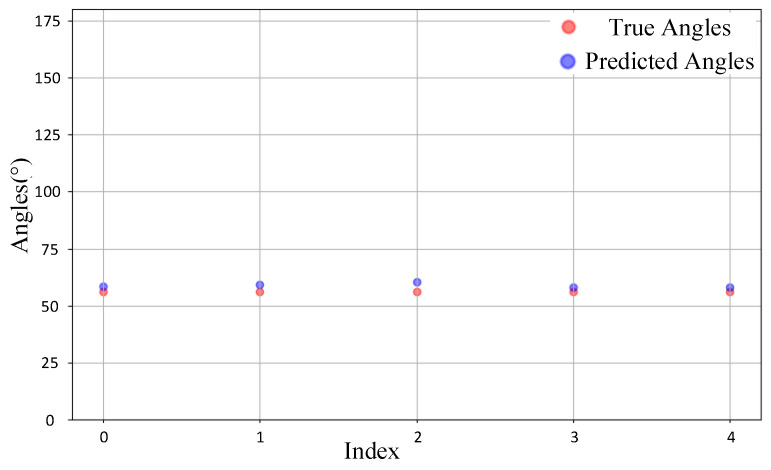
Comparison of 56° samples.

Finally, we tested the model’s prediction ability on non-grid point data, where the true DOA values are not multiples of 5. Specifically, we conducted experiments with true DOA values of 72° and 56°, randomly selecting five IQ data samples from each group for detection. The test results are presented in Figure 16 and Figure 17. From the figure, it can be observed that for these two sets of non-grid point samples, most of the model’s predictions are fairly close to the true DOA values. Although there is a certain degree of deviation, the overall errors remain within an acceptable range. Notably, for the 72° samples, the predicted values exhibit a high degree of overlap with the true values, with only a few samples showing slight underestimation. In contrast, the predictions for the 56° samples display slightly larger deviations, with some predicted values being marginally higher than the true values. Nevertheless, the model still captures the actual angular distribution of the samples reasonably well. These results indicate that the model not only performs effectively with grid point samples but also demonstrates robust predictive capability for the more challenging non-grid point samples.

## 5. Conclusions

This study delves into indoor target DOA estimation and proposes a novel domain-adaptation-based method. Leveraging a minimal amount of labeled data as auxiliary information, the approach first employs a CAE to extract deep features, followed by DANN to align features between the source and target domains. By integrating GRL and MMD loss functions into the network, the method effectively reduces distributional discrepancies between the source and target domains. By using both simulated data and measured data, experimental results demonstrate that, with only a small amount of target domain data incorporated into the source domain, the proposed method outperforms other methods, achieving better DOA estimation performance in both the source and target domains. This method significantly improves target domain prediction performance, effectively addressing the challenge of efficient DOA estimation with limited labeled data in practical applications. In future research, we plan to conduct in-depth investigations into the DOA estimation of moving sources and coherent sources in indoor environments. This will expand the applicability of the proposed method in complex real-world scenarios.

## Figures and Tables

**Table 1:** Run times of different methods.

Methods	Run Time
CAE-DANN	$9.975\times 10^{-3}$ s
DANN	$7.558\times 10^{-3}$ s
CNN	$6.975\times 10^{-3}$ s
MUSIC	$9.662\times 10^{-1}$ s

**Table 2:** CAE hyperparameters.

Parameter	Parameter Value
Optimizer	Adam
Learning Rate	0.001
Hidden Layers	8
Activation Function	ReLU, Sigmoid
Batch Size	256
Epochs	50

**Table 3:** DANN hyperparameters.

Parameter	Parameter Value
Optimizer	Adam
Learning Rate	0.001
FeatureExtractor Hidden Layers	7
LabelPredictor Hidden Layers	3
DomainClassifier Hidden Layers	3
Activation Function	ReLU
Batch Size	256
Epochs	200

**Table 4:** RMSE of different methods.

Methods	Source Domain RMSE	Target Domain RMSE
CAE-DANN	3.156	5.486
DANN (without CAE)	6.651	8.458
CAE-DANN (without MMD)	3.032	8.752
CAE-CNN (without GRL)	2.981	10.976
CNN	2.854	52.466
MUSIC	84.230	92.816

**Table 5:** MAE of different methods.

Methods	Source Domain RMSE	Target Domain RMSE
CAE-DANN	3.591	6.148
DANN (without CAE)	7.275	10.484
CAE-DANN (without MMD)	4.864	11.776
CAE-CNN (without GRL)	3.418	12.257
CNN	3.351	64.527
MUSIC	88.571	124.876

## Data Availability

The raw data supporting the conclusions of this article will be made available by the authors on request.

## Abbreviations

The following abbreviations are used in this manuscript:

LBS	Location-Based Services
VR	Virtual Reality
AR	Augmented Reality
DOA	Direction of Arrival
MUSIC	Multiple Signal Classification
ESPRIT	Estimation of Signal Parameters using Rotational Invariance Techniques
SNR	Signal-to-Noise Ratio
AOA	Angle of Arrival
AOD	Angle of Departure
OMP	Orthogonal Matching Pursuit
LAA	Lens Antenna Array
JADE	Joint Angle and Delay Estimation
TOA	Time of Arrival
RSSI	Received Signal Strength Indicator
SVM	Support Vector Machine
DL	Deep Learning
DNNs	Deep Neural Networks
CNN	Convolutional Neural Network
FSL	Few-Shot Learning
CAE	Convolutional Autoencoder
DANN	Domain-Adversarial Neural Network
GRL	Gradient Reversal Layers
MMD	Maximum Mean Discrepancy
UCA	Uniform Circular Array
LOS	Line-of-Sight
NLOS	Non-Line-of-Sight
MSE	Mean Squared Error
RMSE	Root Mean Squared Error
MAE	Mean Absolute Error
RF	Radio Frequency
